# Using whole genome sequencing to investigate transmission in a multi-host system: bovine tuberculosis in New Zealand

**DOI:** 10.1186/s12864-017-3569-x

**Published:** 2017-02-16

**Authors:** Joseph Crispell, Ruth N. Zadoks, Simon R. Harris, Brent Paterson, Desmond M. Collins, Geoffrey W. de-Lisle, Paul Livingstone, Mark A. Neill, Roman Biek, Samantha J. Lycett, Rowland R. Kao, Marian Price-Carter

**Affiliations:** 1 Institute of Biodiversity, Animal Health, and Comparative Medicine, University of Glasgow, Glasgow, Scotland G61 1QH UK; 2 Wellcome Trust Sanger Institute, Wellcome Genome Campus, Hinxton, Cambridge, UK; 3 TBfree New Zealand, PO Box 3412, Wellington, 6140 New Zealand; 4 AgResearch, Hopkirk Research Centre, Palmerston North, New Zealand; 5 Infection and Immunity Division, The Roslin Institute, University of Edinburgh, Easter Bush, Midlothian, Scotland UK

**Keywords:** Bovine tuberculosis, Whole Genome Sequencing, New Zealand, Substitution rate, Inter-species transmission

## Abstract

**Background:**

Bovine tuberculosis (bTB), caused by *Mycobacterium bovis*, is an important livestock disease raising public health and economic concerns around the world. In New Zealand, a number of wildlife species are implicated in the spread and persistence of bTB in cattle populations, most notably the brushtail possum (*Trichosurus vulpecula*). Whole Genome Sequenced (WGS) *M. bovis* isolates sourced from infected cattle and wildlife across New Zealand were analysed. Bayesian phylogenetic analyses were conducted to estimate the substitution rate of the sampled population and investigate the role of wildlife. In addition, the utility of WGS was examined with a view to these methods being incorporated into routine bTB surveillance.

**Results:**

A high rate of exchange was evident between the sampled wildlife and cattle populations but directional estimates of inter-species transmission were sensitive to the sampling strategy employed. A relatively high substitution rate was estimated, this, in combination with a strong spatial signature and a good agreement to previous typing methods, acts to endorse WGS as a typing tool.

**Conclusions:**

In agreement with the current knowledge of bTB in New Zealand, transmission of *M. bovis* between cattle and wildlife was evident. Without direction, these estimates are less informative but taken in conjunction with the low prevalence of bTB in New Zealand’s cattle population it is likely that, currently, wildlife populations are acting as the main bTB reservoir. Wildlife should therefore continue to be targeted if bTB is to be eradicated from New Zealand. WGS will be a considerable aid to bTB eradication by greatly improving the discriminatory power of molecular typing data. The substitution rates estimated here will be an important part of epidemiological investigations using WGS data.

**Electronic supplementary material:**

The online version of this article (doi:10.1186/s12864-017-3569-x) contains supplementary material, which is available to authorized users.

## Background

Control of a disease in a multi-host system is most efficient when the role of the different hosts is understood [1, 2]. Control of bovine tuberculosis (bTB) in domestic cattle herds is motivated by the zoonotic risk of the causative agent *Mycobacterium bovis*, its impacts on animal productivity, and the benefits of TB-free status in international trade [3]. *M. bovis* infection has been successfully combated in many countries [4–6]. Effective campaigns have relied upon test and slaughter regimes, movement restrictions and abattoir surveillance. Despite success using such regimes, endemic bTB still exists, most notably in areas that have wildlife reservoirs of infection. A broad host range, promoting multi-host bTB systems, is considered to be one means by which *M. bovis* persists in the face of control [7, 8].

In New Zealand, the introduced brushtail possum (*Trichosurus vulpecula*) has long been recognised as an important maintenance reservoir for *M. bovis* [9, 10]. In addition, deer, pigs, and ferrets are thought to act as key spatial and temporal vectors of infection [10]. Control of bTB in cattle herds uses test and slaughter surveillance; more frequent testing and movement control are employed in Vector Risk Areas (VRAs), where the risk of infection from wildlife is highest [11]. Within VRAs, control methods such as trapping and poisoning are primarily aimed at the possum population so as to limit the potential for intra-and inter-species transmission [12]. The incidence of infected cattle herds has been drastically reduced over the last two decades [13] but complete eradication remains elusive, likely as a result of persistent infection in wildlife populations.

Discriminatory molecular typing tools have been extremely helpful in the study of *M. bovis* infection in livestock, informing the tracking of infection [14–16] and improving our understanding of how bTB spreads and persists [17, 18]. Traditionally in New Zealand, Restriction Endonuclease Analysis (REA) typing was used extensively during bTB surveillance. Cattle and wildlife were shown to share the same REA type [19], and importantly, local regionalisation of REA types enabled the distinction between re-infection and introduction [20]. While REA typing is discriminatory, it is technically challenging to perform, interpret and document, and has recently been replaced with Variable Number Tandem Repeat (VNTR) typing [15].

The advent of Next Generation Sequencing has made it increasingly feasible to sequence and compare Whole Genome Sequences (WGS) in order to inform epidemiological analyses. WGS data provide the highest resolution, and therefore discriminatory power, for understanding the sampled system [21, 22]. Recently Glaser et al. [23] used WGS data to distinguish outbreaks carrying identical VNTR types, as well as identifying transmission within and between cattle and deer populations. Similar work in New Zealand has demonstrated the utility of WGS as a robust and highly discriminatory typing method (in prep: Price-Carter et al. 2017). Biek et al. [22] used WGS methods to examine bTB transmission in Northern Ireland, and demonstrated that badgers and livestock living in close proximity shared very similar *M. bovis* strains, suggesting that multiple inter-species transmission events had occurred.

Our research aimed to refine our understanding of the role of wildlife in the transmission and persistence of bTB across New Zealand and estimate the substitution rate of *M. bovis* in this system. Samples taken from infected cattle and wildlife provided *M. bovis* isolates for which WGS data was generated. In agreement with previous knowledge, wildlife species were implicated in the transmission and persistence of bTB infection in the sampled population. We found evidence of multiple inter-species transmission events and estimated their force and direction. Estimating the transmission direction was found to be influenced by the sampling patterns. The availability of WGS data presented the opportunity to evaluate the use of WGS in routine typing. WGS methods were able to discriminate isolates to a finer resolution than REA typing, and there was good agreement between these typing methods. The utility of WGS techniques depends on the frequency with which mutations are fixed within the population. The estimated substitution rate was higher than those previously estimated for *M.bovis.*

## Methods

### Sampling and isolate preparation

**Fig. 1:**
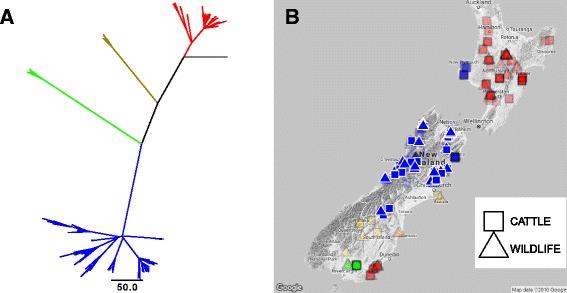
**a** An unrooted maximum likelihood phylogenetic tree built using PHYLIP [61] and rooted using PATH-O-GEN [35]. Assigned clades are coloured accordingly: clade 1 = *blue*, *clade* 2 = red, clade 3 = *gold* and *clade* 4 = *green*. **b** The sampling locations of the isolates are plotted onto a map of New Zealand. Cattle and wildlife isolates are represented by circles and triangles, respectively. Isolates are coloured by their associated *clade* in the phylogenetic tree (**a**). Only isolates from clade 1 (*blue*) were selected for further analysis (*white outline*), faded isolates are those not selected. Isolate locations were jittered to avoid overlapping points. **b** The map underlying b was sourced from Google Maps, 2016 [62]

As part of the routine bTB surveillance in New Zealand, any cattle or wildlife suspected of *M. bovis* infection undergo a post-mortem examination, and if lesions are discovered a selection are investigated using culture and strain typing. Conventional tests (described in *de* Lisle et al. [24]) were used to positively identify *M. bovis* infection. Isolates were REA typed according to previously described methods [16, 25] and cultures were frozen and stored in the strain archive at AgResearch Ltd. Isolates from the archive were selected to provide a representative sample of the *M. bovis* population circulating in cattle and wildlife across New Zealand between 1985 and 2013 (Fig. 1). To create this representative sample, groups of isolates, from cattle and wildlife, of the same or closely related REA type from the same geographical region were selected from the Central North Island region, and the West Coast and Northeast regions of the South Island. The groups were selected to include all of the most frequently isolated REA types.

Selected isolates were re-cultured at AgResearch Ltd. to generate DNA (Deoxyribonucleic Acid) extracts for WGS. Frozen culture stocks were grown to mid log phase (OD600 = 0.4–0.8) in 5 ml of Tween/albumin broth [26] and sub-cultured into 100 ml of the same media for 5 to 11 weeks until the cultures reached stationary phase. Cell cultures were heat killed and stored at −20 °C. Bacterial DNA was specifically separated from the other cellular components with a high salt hexadecyl trimethyl ammonium bromide (CTAB) extraction. DNA was then extracted with a chloroform/isoamyl alcohol method and quantified using Invitrogen Qubit fluorometry.

A selection of 90 DNA isolates were sequenced at the University of Glasgow Polyomics Facility using an Illumina MiSeq platform that produced 2 × 300 bp paired end reads per isolate. 17 additional isolates were sequenced at New Zealand Genomics Ltd. on a MiSeq platform that produced 2 × 250 bp paired end reads. The remaining isolates (*n* = 204) were sequenced at the Wellcome Trust Sanger Institute using an Illumina HiSeq that produced 2 × 100 bp paired end reads.

### Processing sequencing data

The raw reads for each isolate were examined using FASTQC (v0.11.2 - Andrews [27]) to identify poor quality ends that were then trimmed using PRINSEQ (v0.20.4 - Schmieder & Edwards [28]). If adaptor sequences were present these were removed using TRIMGALORE (v.0.4.1 - Krueger [29]). Each isolate’s trimmed reads were aligned to the *M. bovis* reference genome, AF2122/97 [30], using the freely available Burrows-Wheeler Alignment tool [31, 32]. The mean coverage (sites with Read Depth (DP) ≥ 20) for the isolates was 99% (2.5% Lower: 96.9, 97.5% Upper: 99.8).

Site information across the isolates was collated to allow the quality of individual sites to be assessed. Sites that fell within Proline-Glutamate (PE) and Proline-Proline-Glutamate (PPE) genes or annotated repeat regions were removed (Sampson [33]). Thereafter only Variant Positions (VPs), sites for which at least one of the isolates showed variation against the reference genome, were retained.

High quality sites were selected for subsequent analyses based on the Mapping Quality (MQ), High Quality base Depth (HQDP) and Read Depth (DP). Filters were designed using MQ, HQDP, DP, in addition to the support for the allele called (SUP), the site coverage across the isolates (COV), and the number of positions that separated SNPs (PROX). A filter sensitivity analysis was conducted in order to establish an optimal filter combination (See Additional file 1: Supplementary text 1.1). The following filters were selected: MQ ≥ 30, HQDP ≥ 4, DP ≥ 30, SUP ≥ 0.95, COV ≥ 0.7, and PROX = 10.

### Isolate selection

An early examination of the WGS data revealed that, although most isolates with the same Restriction Endonuclease Analysis (REA) type were very similar, several isolates were quite distinct from the others with the same REA type. These “outliers” were further investigated to determine whether they were mislabelled. Although it was not possible to re-examine these isolates with the REA typing method, potentially mislabelled samples were further examined with Variable Number Tandem Repeat (VNTR) assays. Specific REA types are known to be associated with specific VNTR types. VNTR assays were conducted (described in [15]) using a subset of VNTR loci that were likely to discriminate the isolates in question. For controls, a selection of isolates with similar sample numbers to the questionable isolates was also re-examined. If the determined VNTR types differed from what would have been expected based on previous analyses of these types [15], the isolate was considered to have been mislabelled. 15 of the 28 (14 suspects and 14 controls) isolates examined had VNTR loci that differed from what was expected. These 15 mislabelled isolates were removed from any further analyses (See Additional file 1: Supplementary text 1.2), leaving 296 isolates for further investigation.

Using the 296 isolates, a maximum likelihood phylogenetic tree was constructed in the program PHYLIP (v3.695 - Felsenstein [34]) and rooted using the program PATH-O-GEN (v1.4 - Rambaut [35]). For each isolate the sampling location (including latitude and longitude) and year (of sample submission), sampled species, and REA type were available. Using the maximum likelihood tree and the available sampling information, a selection of spatially and temporally associated isolates were chosen from within clade 1 (Fig. 1a).

**Fig. 2:**
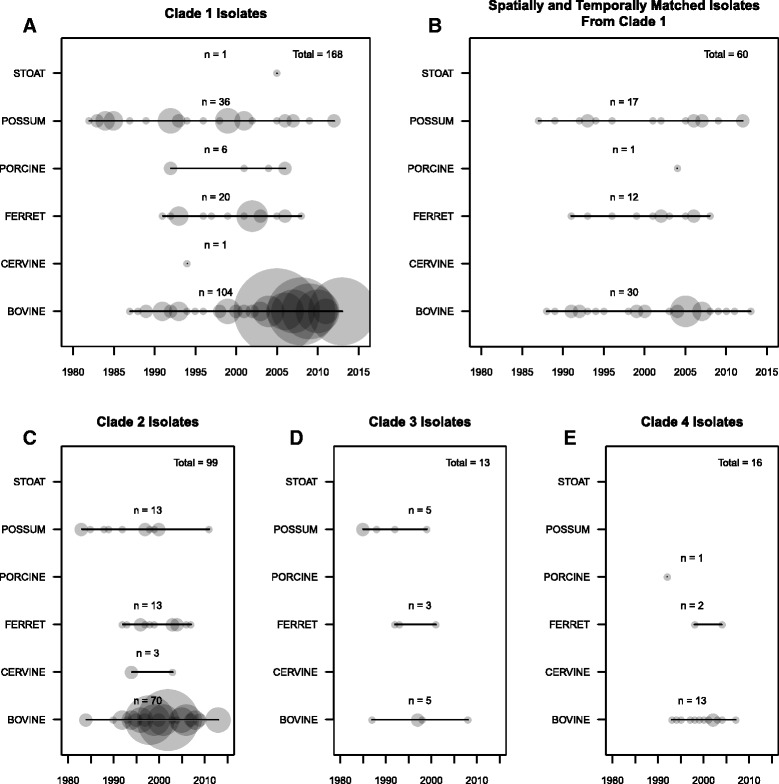
Five plots illustrating the temporal range associated with each sampled host species for all the isolates in the different clades (1 (**a**), 2(**c**), 3(**d**), and 4(**e**)), and the spatially and temporally associated isolates from clade 1 (**b**). The size of each point is scaled by the number of isolates that were taken from the given species in the given year

Although a large number of isolates were available for the current analyses, these isolates fell within highly distinct clades. Isolates from a single clade were selected to ensure a relatively recent common ancestor to the isolates analysed, and limit the effects of biases introduced by examining genetically distinct groups. Unique pairs of cattle and wildlife isolates were chosen from within strict spatial (40 km) and temporal (+/− 3 years) limits to reduce the impact of potential temporal and spatial sampling biases (Figs. 1b and 2). The spatial and temporal thresholds were chosen so as they were the minimum values necessary to retain a large enough sample size for further analyses. Using the spatial and temporal thresholds described, only Clade 1 had enough spatially and temporally associated isolates to warrant further analyses.

### Clustering of inter-isolate genetic distances

The available data for the isolates-REA type, sampling location (district where the sampling took place) and sampled host - were used to define groups of isolates and the within- and between-group genetic distances were examined to determine whether there was an association. The concatenated sequence of VPs of each isolate was compared to one another to generate an inter-isolate genetic distance (using the p-distance-defined as the proportion of the sites that differ between two sequences).

The observed difference between the mean intra- and inter-group genetic distances was calculated where the groups were defined, separately, by host species sampled, sampling location and REA type. To determine whether each observed difference could have arisen by chance alone, the isolate data was shuffled and the difference re-calculated. The shuffling was repeated 10,000 times to generate null distributions of observed differences. The associations were considered significant if the observed metric fell outside the lower (2.5%) and upper (97.5%) quantiles of the null distribution. Importantly, any species signature is likely to be nested within a spatial one, since regional localisation of bTB is known. To account for this, only comparisons that were between isolates sampled in the same district were included in the clustering analyses using the host species sampled.

### Phylogenetic Analyses

The Bayesian Evolutionary Analysis by Sampling Trees (BEAST v1.8.4 - Drummond & Rambaut [36]) software was used for a phylogenetic analysis of the isolates’ sequences combined with their sampling years. BEAST was used to estimate the phylogenetic tree topology, substitution rate and date of the Most Recent Common Ancestor (MRCA) for the sampled *M. bovis* population. A BEAST analysis requires the existence of a clock-like substitution process. Additional analyses, as conducted by Firth et al. [37], were used to examine whether a clock-like process could have produced the inter-isolate variation (See Additional file 1: Supplementary text 1.3).

Models selected in a BEAST analysis may significantly impact the results. Care must be taken to select appropriate models for the substitution process [38] and the underlying population dynamics [39]. A series of BEAST analyses were completed in a hierarchical fashion to explore the different models available; for each analysis a chain length of 500,000,000 steps, sampled every 50,000 steps, was used and three replicates were completed. Following the removal of a 10% burn-in, the posterior distributions were examined to determine which structure of BEAST analysis best described the isolate data. Different analyses were compared based upon the log likelihood scores, model convergence and posterior support of parameters (assessed using TRACER v1.6 [40]), path sampling and stepping stone analyses (See Additional file 1: Supplementary text 1.4). In addition, the biological feasibility of the results was examined for each analysis.

The selected BEAST analysis used the Hasegawa-Kishino-Yano (HKY) substitution model, a relaxed clock model, drawing from an exponential distribution, and the Gaussian Markov Random Field (GMRF) Bayesian Skygrid population model. The HKY substitution model allows variable base frequencies, transition and transversion rates to be estimated [41]. A relaxed clock model enabled the estimated substitution rate to vary across the branches of the phylogenetic tree; the extent of this variation was modelled using an exponential distribution. The GMRF Skygrid model is a flexible model that is able to estimate changing population dynamics over the course of a phylogenetic history [39]. In a BEAST analysis, population dynamics are estimated based on the structure of the phylogenetic tree according to coalescent theory [42].

An additional Discrete Ancestral Trait Mapping (DATM) analysis [38, 43] for two states was implemented in the BEAST analysis. According to the host species sampled, isolates were assigned either a cattle or wildlife state. Based upon the states of the tips of the phylogenetic tree (the isolates) the DATM estimates the ancestral states in the phylogeny, and as such the most likely sources of infection within the sampled *M. bovis* population. A comparison was made between a symmetric and asymmetric DATM analyses in BEAST using the spatially and temporally matched isolates. The former symmetric analysis refers to the state transition matrix being symmetric; this analysis estimates a single parameter (in a two state analysis), the transmission rate of the pathogen from one state to another. The asymmetric analysis has two inter-state transmission parameters and as such can be used to determine whether there is a directional bias in the exchange; is the pathogen jumping from one population into another more often than in the other direction?

The influence of the selection of prior distributions for the parameters estimated in the BEAST analyses, described above, was investigated by running an analysis where the data were removed and only the prior distributions sampled. It was shown that the selected prior distributions were conservative and that the data provided a strong signal for the parameter estimations of our model (See Additional file 1: Supplementary text 1.5).

## Results

### Structure in the sampled M. bovis population

There were four recognisably distinct clades formed by the 321 isolates sampled in New Zealand that were regionally localised (Fig. 1). A total of 3449 VPs were found. Long distance translocation and establishment of new foci of infection was evident, when the genetic structure of the population was considered. Clade 2 (Fig. 1 – red), although mostly found in New Zealand’s North Island, has an established foci of infection involving both cattle and wildlife on the South Island. Clade 1 (Fig. 1 – blue) isolates were mostly situated on the South Island of New Zealand, providing a densely sampled genetically similar set from which to select the spatially and temporally associated isolates for further analysis (Figs. 1 and 2). Clade 3 (Fig. 1 – gold) included eight wildlife and five cattle isolates, found across a broad spatial range in the southwest of New Zealand’s South Island. Clade 4 (Fig. 1 – green) also included thirteen cattle and three wildlife isolates, which were sampled from two locations <20 km apart in the south of New Zealand’s South Island.

### Clustering of inter-isolate genetic distances

**Fig. 3:**
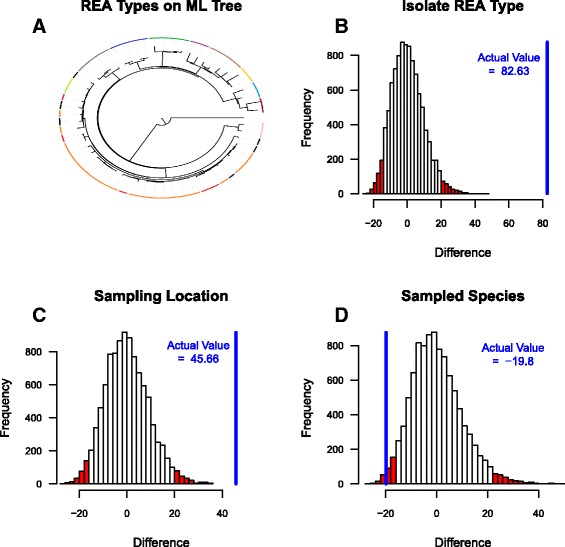
Clustering in the inter-isolate genetic distance distribution for the spatially and temporally matched isolates from clade 1. **a** A Maximum Likelihood phylogenetic tree generated using PHYLIP; coloured bars are used to highlight isolates that have the same REA type (note that REA types that are only represented by one isolate are colour in black). **b**, **c**, and **d** Three plots showing how the observed difference between the mean inter- and mean intra-group genetic distances, when isolate groups were defined by REA, Sampling Location, and Species (**b**, **c**, and **d**, respectively) compared to null distributions of differences calculated on shuffled sequences. The sampling location was defined as the region where sampling occurred. The difference was calculated for 10,000 independently shuffled sets. Only the spatially and temporally matched isolates from clade 1 were used in this clustering analysis. The blue line shows the observed difference between mean inter- and mean intra-group genetic distances. The area outside of the lower (2.5%) and upper (97.5%) bounds of the null distribution are coloured in red

The inter-isolate genetic distance distribution of the spatially and temporally associated isolates from clade 1 was examined. Isolates of the same REA type were, on average, more genetically similar than those of different types. This difference was reflected in lower average within- than between-group genetic distances, when groups were defined by REA types (Fig. 3b). In addition, diversity was evident in the within-group distances demonstrating the added resolution of WGS data. The observed difference between the mean inter- and intra-group genetic distances was unlikely to have arisen by chance when the isolates were grouped by their REA type, sampling location or sampled species (Fig. 3b and c). In contrast to the lower within- than between-group genetic distances observed when groups were defined by REA type or sampling location (as was demonstrated by the positive observed difference (Fig. 3b and c)), when groups were defined by the host species sampled, the within-group distances were higher than the between-group distances (Fig. 3d). These higher within-group distances resulted in a negative observed difference, which was unlikely to have arisen by chance as it fell just outside the 95% bounds of the generated null distribution. This negative difference may be caused by lower within-outbreak distances resulting from sampling local outbreaks (involving cattle and wildlife) that are separated in space.

### Substitution rate estimation

**Fig. 4:**
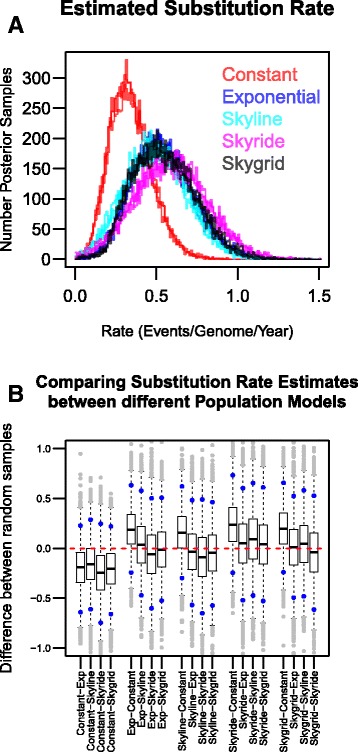
The estimated substitution rate of the sampled *M. bovis* population. **a** The sampled (*n* = 9000, 10% burn-in removed) posterior distributions of the substitution rate estimated by BEAST analyses using different population models. Each analysis in BEAST was repeated 3 times and replicates plotted with the corresponding colour for the population model. **b** Pairwise comparisons of the posterior distributions resulting from analyses based upon different population models. Each boxplot summarises the distribution of differences produced by calculating the difference between 10,000 random samples of the posteriors being compared. The blue points represent the upper and lower bounds of distribution of differences. Outliers of the difference distributions are coloured in *grey*

Using a bootstrapping procedure the posterior distributions resulting from BEAST analyses incorporating different population models were compared (Fig. 4b). For each pairwise posterior comparison, a distribution of differences was generated by calculating the difference between single point estimates, that were sampled proportionately from each of the two posterior distributions. If similar distributions are compared using this pairwise comparison, the calculated differences between point estimates drawn randomly from each distribution will be close to zero. The paired posterior distributions were not significantly different; the distribution of calculated differences resulting from each pairwise comparison overlapped with zero. The Skygrid population model, which had a high likelihood in the model selection procedures (See Additional file 1: Supplementary text 1.4) and agreed well with the other population models used (Fig. 4), estimated the substitution rate of the sampled *M. bovis* population to be 0.53 (2.5% Lower: 0.22, 97.5% Upper: 0.94) events per genome per year.

Using the Skygrid population model, the MRCA to the sampled *M. bovis* population was estimated to have been circulating in 1859 (2.5% Lower: 1525, 97.5% Upper: 1936). Binney et al. [44] recently established that a large number of cattle were imported into New Zealand in the 1860s, mostly originating from Australia and the United Kingdom. The structure of clade 1 and of the full maximum likelihood phylogeny (Fig. 1a) aren’t indicative of a single introduction event into New Zealand. The sampling window used in this study was narrow, relative to the phylogenetic history of the sampled population. Via simulation it was shown that estimates of the substitution rate were robust to the shortening of the sampling window; estimates increasingly lacked precision but retained accuracy (data not shown).

### Ancestral traits analysis

**Fig. 5:**
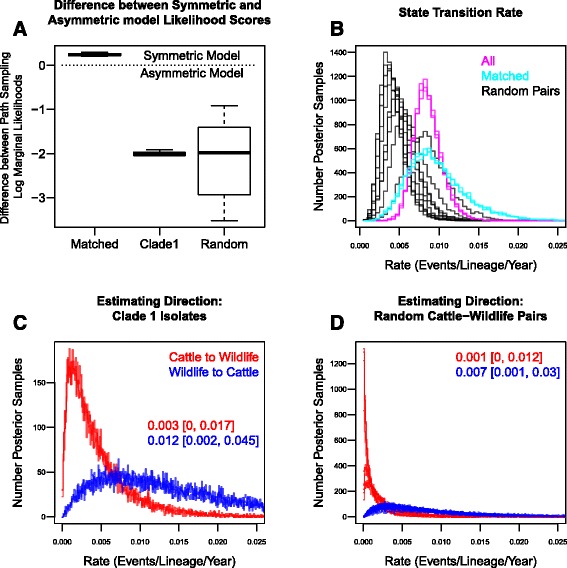
The state transition rates estimated by a discrete traits analysis in BEAST on isolates selected from clade 1. States were defined as either Cattle or Wildlife. BEAST analyses were completed using all the clade 1 isolates (3 replicates), only spatially and temporally matched isolates (3 replicates) and 30 randomly matched cattle and wildlife isolates (10 replicates). **a** A box plot of the difference between the likelihoods (estimated using path sampling) of the symmetric and asymmetric models for the different sampling sets. The symmetric model was favoured for the matched isolates and the asymmetric model for the analyses based on all the clade 1 isolates and randomly matched cattle and wildlife. **b** The sampled (*n* = 10,000) posterior distributions of the estimated overall transition rate between Cattle and Wildlife based on the three sampling sets. Plots (**c** and **d**) show the posterior distributions of the transition rates from Cattle to Wildlife (*Red*) and Wildlife to Cattle (*Blue*) resulting from BEAST analyses completed on the clade 1 isolates and the randomly matched isolates. The median and 95% Credible Intervals are stated for the distributions

Different selections of isolates from clade 1 (all isolates, temporally and spatially matched isolates, or 30 random cattle and wildlife pairs) were used in separate DATM BEAST analyses. According to the path sampling likelihood values, the symmetric model (equal rates from cattle to wildlife and vice versa) was favoured for the matched isolates (Fig. 5a). The asymmetric (different rates) was favoured when the DATM analysis was based on all the clade 1 isolates or the randomly paired isolates (Fig. 5a). The DATM analyses were able to provide an estimate for the overall state transition rate (Fig. 5b). For the analyses based on all the clade 1 isolates or randomly matched isolates the asymmetric model provided directional estimates of the state transition rates (Fig. 5c and d). Using all isolates or the random cattle and wildlife pairs, a dominant direction of transmission from wildlife to cattle was estimated (Fig. 5c and d). Using the spatially and temporally matched isolates the symmetric model out-performed the asymmetric model and directional estimates weren’t available. The difference in the support for the symmetric versus asymmetric model was a result of the isolates selected and therefore shows a strong influence of sampling. Without knowing which sample set is most representative it is difficult to have confidence in the directional state transition rates.

## Discussion

The current research suggests that *M. bovis* infection was being transmitted between the sampled wildlife and cattle populations. In Northern Ireland, where the role of badger populations is under investigation, WGS data has been used in an attempt to elucidate the mechanisms of persistence of bTB in cattle herds [22, 45]. High genetic similarity suggested recent transmission links between badger and cattle populations. Similarly, Glaser et al. [23], used WGS data to reveal exchange within and between cattle and deer populations in Minnesota.

By the end of June 2015, there were 39 infected cattle herds in New Zealand [11]. Whilst cattle movements remain a recognised cause of newly detected herd infections, wildlife are thought to be the main contributors. In the current research, isolates originating from cattle and wildlife sources were indistinguishable suggesting a high degree of exchange. A high degree of exchange was supported by the estimations of an overall inter-species rate. With New Zealand’s low prevalence of bTB in livestock, despite being unable to estimate inter-species transmission direction in the current research, it would seem highly likely that wildlife populations are acting as maintenance reservoirs and as such should remain the target of the eradication campaign.

When investigating any epidemiological process using genetic data of a pathogen, the relative speed of that epidemiological process compared to the rate of change of the sampled pathogen must be considered [46]. Ideally, the sampling of a system of interest should reflect the underlying epidemiological processes, and not produce additional noise or biases. For example, if isolates are too distantly related (both genetically and epidemiologically) noise may dominate the signal of the epidemiological events of interest, making the estimation of these events difficult. In addition, it is important that the sampling of a system of interest is designed such that potential epidemiological events are likely to be captured and not masked by additional noise. For example, if highly distinct isolates result from the sampling, too many of the epidemiological events of interest may have occurred in the shared history of the isolates, making the estimation of these events difficult.

Here, the inter-species transmission rate was estimated. The difficulty encountered when estimating the direction of interspecies transmission may be a reflection of a high rate of exchange of *M. bovis* between cattle and wildlife estimated using a slowly evolving pathogen sampled from a broad genetic distribution.

The role of wildlife in the maintenance of bTB in New Zealand could provide an explanation for why the substitution rate estimated here was relatively high, in comparison to previously published estimates of the substitution rate for the *M. tuberculosis* complex (Table 1). This difference will enhance the utility of genomic data for routine epidemiological investigations because it will allow for better estimates of the time of introductions of new infections into herds and wildlife populations and thus aid in the identification of likely sources of infection.

**Table 1:** A comparison between estimates of the substitution rates (events per genome per year) taken from WGS analyses on *M. tuberculosis* and *M. bovis*, with the results of this study inserted in the final row

Published Source	Bacteria Species	Mean/Median	Lower	Upper	Host Sampled	Country
Walker et al., 2013	*M. tuberculosis*	0.5	0.3	0.7	Human	UK
Bryant et al.,[63]	*M. tuberculosis*	0.3	NA	NA	Human	Netherlands
Roetzer et al., [21]	*M. tuberculosis*	0.4	0.3	0.7	Human	Germany
Biek et al., [22]	*M. bovis*	0.15	0.04	0.26	Cattle/Badger	UK
Trewby et al. 2015	*M. bovis*	0.2	0.1	0.3	Cattle/Badger	UK
Current Research	*M. bovis*	0.53	0.22	0.94	Cattle/Possum	New Zealand

Most brushtail possums suffer an extensive *M. bovis* infection if exposed, and many will die within 6 months [47, 48]. In contrast, the majority of humans, cattle, and badgers suffer a localised latent TB infection [49–51]. Given that herd breakdowns in New Zealand are thought to be mainly the result of spill-over events from wildlife vectors [11, 20], the higher levels of replication during the more extensive infection in possums could result in an increased accumulation of mutations for the sampled *M. bovis* population.

Colangeli et al. [52] demonstrated that the likely lower rates of replication occurring during latent *M. tuberculosis* infection, in humans, resulted in significantly lower accumulation of mutations when compared to active infection. This research supports the theory that replication rates impact substitution rates and is consistent with other observations on host-level variability [53]. However, Ford et al. [54] were unable to find an effect of latency on the substitution rate of *M. tuberculosis* in infected Macaque monkeys, in an experimental setting, and so this area requires further study. Alternatively, the higher substitution rate could be the result of a lineage specific trait, such differences have been demonstrated in *M. tuberculosis* [55, 56].

The patterns of sampling and their influence on results of any analysis are an important consideration. Broad credible intervals were estimated around the substitution rate and date of the MRCA for the sampled population. The isolates analysed in the current research were sampled between 1987 and 2013; relative to the estimated root height (1859 [1525, 1936]), this sampling window is narrow. *M. bovis* is likely to have been circulating in New Zealand since the mid-1800s [12], therefore sampling early in this outbreak wasn’t possible. Analyses based on simulated epidemics sampled using an increasingly late and narrow window, demonstrated that a narrow sampling window had a pronounced effect on the precision of estimates but, importantly, little effect on the accuracy of parameter estimation in BEAST (data not shown).

In the DATM analysis a temporal bias was evident in the original set of clade 1 isolates (Fig. 2), with dense sampling of wildlife in early years and of cattle in later years, resulted in a dominant direction of spread from wildlife to cattle being estimated. Using the current data it wasn’t possible to determine whether this dominance exists, and the sampling patterns are a true reflection of New Zealand’s bTB system, or the directionality observed was an artefact of the sampling patterns.

The WGS data provided added resolution to the examination of bTB in New Zealand, distinguishing isolates sharing an identical REA type (Fig. 3a). The declining cost, added resolution, good agreement with REA typing, and evidence of a strong spatial signature all act to endorse the use of WGS typing in routine surveillance.

The utility of any typing method lies in its molecular clock speed; too quick and noise masks important events, too slow and important events could be missed [57, 58]. Both human- and bovine-TB are caused by slowly evolving, genetically conserved, bacteria [17, 30]. With such a recognisably slow rate of change it is unlikely that infection dynamics within or between individuals will result in significant genetic signatures. In contrast herd level signatures are likely to be present and of use in routine surveillance that targets herds [22, 23, 46].

## Conclusions

In the current research it was shown that knowledge of epidemiology combined with WGS data can provide a means for in-depth investigations of bTB dynamics, shedding light on important and as yet unquantified features, such as the extent of inter-species transmission and the substitution rate. A caveat though, the influence of the sampling strategy used, should be thoroughly examined as to its potential impact on any findings. Targeted control of wildlife populations is part of New Zealand’s eradication strategy [12] and wildlife were implicated in the current research. Identifying local persistence or introduction is the focus of bTB surveillance in New Zealand and regional localisation of isolates makes this possible. WGS data, despite the low substitution rate of *M. bovis,* adds resolution, decreasing the scale at which persistence versus introduction can be evaluated. In addition, an estimate of the substitution rate of *M. bovis* in New Zealand, however broad, will inform these evaluations. For routine surveillance, the resolution gained by using WGS data must be weighed against any increased costs, a decision that will be aided by the decreasing price of sequencing technologies.

## Additional file

Additional file 1: Supplementary text 1.1: Filter Sensitivity Analysis. Supplementary text 1.2: Investigating Highly Distinct Isolates. Supplementary text 1.3: Temporal Signal. Supplementary text 1.4: Hierarchical Model Selection. Supplementary text 1.5: Influence of the Priors. **Table S1.** Results from investigating highly distinct isolates. **Table S2.** Results from hierarchical model selection. (DOCX 36 kb)

## ᅟ

bTB: Bovine tuberculosis
COV: Site Coverage across the isolates
CTAB: Hexadecyl trimethyl ammonium bromide
DATM: Discrete Ancestral Trait Mapping
DNA: Deoxyribonucleic Acid
DP: Read Depth
GMRF: Gaussian Markov Random Field
HKY: Hasegawa-Kishino-Yano
HQDP: High Quality base Depth
MQ: Mapping Quality
MRCA: Most Recent Common Ancestor
PE: Proline-Glutamate
PPE: Proline-Proline-Glutamate
PROX: The number of positions that separated SNPs
REA: Restriction Endonuclease Analysis
SNP: Single Nucleotide Polymorphism
SUP: Support for the allele called
VP: Variant Position
VNTR: Variable Number Tandem Repeat
VRA: Vector Risk Area
WGS: Whole Genome Sequenced
