# Supplementary material for: Using whole genome sequencing to investigate transmission in a multi-host system: bovine tuberculosis in New Zealand
Source: BMC Genomics. 2017 Feb 16;18:180. doi: 10.1186/s12864-017-3569-x (PMC5314462; doi:10.1186/s12864-017-3569-x)
Supplement: Additional file 1: — Supplementary text 1.1: Filter Sensitivity Analysis. Supplementary text 1.2: Investigating Highly Distinct Isolates. Supplementary text 1.3: Temporal Signal. Supplementary text 1.4: Hierarchical Model Selection. Supplementary text 1.5: Influence of the Priors. Table S1. Results from investigating highly distinct isolates. Table S2. Results from hierarchical model selection. (DOCX 36 kb) [file 12864_2017_3569_MOESM1_ESM.docx]

# Supplementary text

## Supplementary text 1.1: Filter Sensitivity Analysis

The VPs for each isolate were filtered according to their specific MQ, HQDP, DP and allele support (SUP, the proportion of reads mapped to the position of interest that support the allele called for that position). In addition, the proportion of isolates which had sufficient quality at each VP (COV) and the number of positions that SNPs had to be apart (SNP proximity – PROX) were used as filters. A sensitivity analyses was conducted in order to establish an optimal filter combination.

For each isolate the sampling year, location (region, district, and latitude and longitude data of sampling location), sampled species, and the REA (Restriction Endonuclease Analysis) type was available. This isolate information was used to inform the selection of thresholds (for the filters described above), based upon the assumption that these data should, to some degree, explain the variation observed in the inter-isolate genetic distance distribution.

Inter-isolate comparisons were made using the discrete (district, region, species, and REA) and continuous (spatial and temporal distances) data that were then fitted to the corresponding inter-isolate genetic distance using a random forest (RF) regression model [61]. The RF model was fitted in the statistical programming environment R (3.0.1).

A filter combination was selected by maximising the variation explained using the fitted RF model. The following filters were selected: MQ≥30, HQDP≥4, DP≥30, SUP≥0.95, COV≥0.7, PROX=10; VPs were filtered based on this filter combination to create a concatenated sequence FASTA file.

## Supplementary text 1.2: Investigating Highly Distinct Isolates

A selection of 14 isolates that were highly genetically distinct from isolates sharing the same Restriction Endonuclease Analysis (REA) type were investigated using VNTR assays (according to Price-Carter et al. 2011). This investigation aimed to determine whether these isolates may have been mislabelled. Specific VNTR loci are known to be associated with different REA types, these VNTR loci were used in assays for the selected isolates. In addition, 14 controls were selected that had similar isolate numbers. Table 1 shows the results of the VNTR assays, the observed VNTR types are compared to those expected given the stated REA type of the isolate.

## Supplementary text 1.3: Temporal Signal

The existence of a temporal signal in the data was examined. A linear regression was conducted on the sampling year against the root-to-tip distance for the temporally and spatially matched isolates from clade 1 and the reference (to aid with the tree rooting). A significant relationship was observed (p-value=0.018) with an R^2^ value of 0.075. The estimated substitution rate, 0.53 (2.5% Lower: 0.22, 97.5% Upper: 0.94) events per genome per year, was compared to an analysis using the same model structure but the associated sampling dates were randomly shuffled (suggested in Firth et al. 2010). The sampling date shuffling was repeated 10 times and BEAST was used to estimate the substitution rate. A substitution rate of 2.85 * 10^-5^ (2.5% Lower: 2.81 * 10^-6^, 97.5% Upper: 1.24 * 10^-4^) events per genome per year was estimated using the shuffled data. These estimations are significantly lower than those estimated on the unshuffled data. The difference between the substitution rates based upon shuffled and true data, in addition to the significant relationship observed between the root-to-tip distances and sampling year, support the presence of a temporal signal to inform the estimation of a substitution rate for the sampled population.

## Supplementary text 1.4: Hierarchical Model Selection

Within BEAST there are a number of different models that can be used to represent the evolutionary processes of the sampled population. A series of analyses were completed, in BEAST, to explore the range of models available. A decision tree approach was employed; 1) a range of substitution models were examined whilst using the simplest clock and population models, 2) once the substitution model had been selected, the available clock models were evaluated using the selected substitution model and the simplest population model, and 3) lastly, the different population models were investigated in combination with the selected substitution and clock models.

Each BEAST analysis in the decision tree was repeated three times. At each level (1, 2, and 3) in the tree the analyses were compared based upon log likelihood scores, model convergence, posterior support of parameters, path sampling and stepping stone analyses. Table 2 shows the structure of the decision tree and the models that were selected.

## Supplementary text 1.5: Influence of the Priors

The models selected in a BEAST analyses have set parameters whose estimation requires the specification of prior distributions. Here prior knowledge about the sampled population was used to inform the BEAST analyses.

The prior distributions for the final model were specified as follows:

HKY substitution model

- Allele frequencies were estimated using the sequence data provided
- The transition-transversion parameter prior distribution was a Log Normal distribution with a log(mean) of 1 and log(SD) of 1.25
- Four categories, modelled with a gamma distribution, were included to allow the substitution rate to vary across sites. The shape parameter for the gamma distributions was estimated from an Exponential distribution with a mean of 0.5

Clock Model

- A relaxed clock model drawing from an Exponential distribution with a mean of 0.005 (events per site per year) was used.

Population Model

- The Skygrid model uses a smoothing parameter to avoid large jumps in the population size estimated based upon the structure of the phylogenetic tree. A Gamma distribution (shape and scale values equal to 0.001 and 1000, respectively), was used as the prior distribution for the smoothing parameter.

The prior distributions specified define the space used to estimate the parameters of interest. It is important to investigate the extent to which the estimates resulting from a BEAST analysis are influenced by the specification of the prior distributions. A BEAST analysis, using the prior specifications defined above, was completed using only the sampling year data (for the spatially and temporally matched isolates from clade 1). This prior sampling analysis estimated a substitution rate of 0.004 (97.5% Lower: 0, 2.5% Upper: 0.19) events per site per year. This estimate is orders of magnitude higher than the substitution rate estimated when the genetic data were included. A right-skew on the true substitution rate estimates was not evident; despite the broad and inaccurate prior distribution specified, there was enough of a signal in the data to estimate the substitution rate.

| **Isolate ID** | **Type** | **REA** | **Exp etrD** | **Obs etrD** | **Exp DR1** | **Obs DR1** | **Exp NZ2** | **Obs NZ2** | **Exp Q26** | **Obs Q26** | **Exp Q11a** | **Obs Q11a** | **Exp DR2** | **Obs DR2** | **Exp**  **3232** | **Obs 3232** |
| --- | --- | --- | --- | --- | --- | --- | --- | --- | --- | --- | --- | --- | --- | --- | --- | --- |
| 2122/97 | Control | 21 | 5 | 5 | 5 | 5 | - | - | - | - | - | - | - | - | - | - |
| 202 | Suspect | 1/6 | 4 | 5 | 5 | 5 | - | - | - | - | - | - | - | - | - | - |
| 203 | Suspect | 62 | 4 | 4 | 3 | 5 | - | - | - | - | - | - | - | - | - | - |
| 204 | Control | 62 | 4 | 4 | 3 | 3 | - | - | - | - | - | - | - | - | - | - |
| 205 | Suspect | 62 | 4 | 3 | 3 | 5 | - | - | - | - | - | - | - | - | - | - |
| 206 | Suspect | 1/6 | 4 | 4 | 5 | 3 | - | - | - | - | - | - | - | - | - | - |
| 207 | Control | 62 | 4 | 4 | 3 | 3 | - | - | - | - | - | - | - | - | - | - |
| 210 | Suspect | 21 | 5 | 4 | 5 | 3 | - | - | - | - | - | - | - | - | - | - |
| 211 | Suspect | 300 | 3/4 | 4 | 5 | 3 | 5 | 5 | 4 | 4 | 9 | 9 | 15 | 15 | 6 | 8 |
| 212 | Control | 62 | 4 | 4 | 3 | 3 | 5 | 5 | 4 | 4 | 9 | 9 | 15 | 15 | 8, 9 | 8 |
| 213 | Suspect | 19 | 3 | 4 | 5 | 5 | - | - | - | - | - | - | - | - | - | - |
| 122 | Suspect | 21 | 3/5 | 4 | 5 | 5 | 5 | 5 | 4 | 4 | 9 | 9 | 15 | 15 | 5,7,8 | 8 |
| 123 | Control | 62 | 4 | 4 | 3 | 3 | 5 | 5 | 4 | 4 | 9 | 9 | 15 | 15 | 8, 9 | 8 |
| 185 | Suspect | 93 | 4 | 5 | 6 | 5 | 5 | 5 | 4 | 4 | 10 | 9 | 13 | 15 | 9 | 8 |
| 186 | Control | 62 | 4 | 4 | 3 | 3 | 5 | 5 | 4 | 4 | 9 | 9 | 15 | 15 | 8, 9 | 8 |
| 267 | Suspect | 16 | 3/4 | 5 | 5 | 5 | 5 | 5 | 4 | 4 | 6/9 | 9 | 15 | 15 | 7,8,9 | 8 |
| 268 | Control | 12 | 4 | 4 | 5 | 5 | 5 | 5 | 1,3,4 | 3 | 9 | 9 | 15 | 15 | 8,9,10,12 | 8 |
| 314 | Suspect | 6 | 4 | 4 | 5 | 3 | 5 | 5 | 4 | 4 | 9 | 9 | 15 | 15 | 6 | 8 |
| 315 | Suspect | 62 | 4 | 4 | 3 | 5 | 5 | 5 | 4 | 4 | 9 | 9 | 15 | 15 | 8, 9 | 6 |
| 316 | Control | 6 | 4 | 4 | 5 | 5 | 5 | 5 | 4 | 4 | 9 | 9 | 15 | 15 | 6,4,8 | 6 |
| 9 | Control | 113 | 4 | 4 | 6 | 5 | 5 | 5 | 4 | 3 | 10 | 10 | 12 | 12 | 10 | 10 |
| 10 | Control | 115 | 4 | 4 | 5 | 5 | 5 | 5 | 3 | 3 | 10 | 10 | 12 | 12 | 7 | 7 |
| 11 | Control | 115 | 4 | 4 | 5 | 5 | 5 | 5 | 3 | 3 | 10 | 10 | 12 | 12 | 7 | 7 |
| 12 | Control | 115 | 4 | 4 | 5 | 5 | 5 | 5 | 3 | 3 | 10 | 10 | 12 | 12 | 7 | 7 |
| 18 | Control | 115 | 4 | 4 | 5 | 5 | 5 | 5 | 3 | 3 | 10 | 10 | 12 | 12 | 7 | 7 |
| 19 | Suspect | 113 | 4 | 4 | 6 | 5 | 5 | 5 | 4 | 3 | 10 | 10 | 12 | 12 | 10 | 7 |
| 20 | Suspect | 115 | 4 | 4 | 5 | 5 | 5 | 5 | 3 | 3 | 10 | 10 | 12 | 12 | 7 | 10 |
| 21 | Control | 151 | 4 | 4 | 5 | 5 | 6 | 6 | 4 | 4 | 10 | 10 | 12 | 12 | 7 | 7 |

Table S1: The expected and observed VNTR loci present for a selection of 14 suspect and 14 control isolates. Where the observed doesn’t match the expected VNTR loci for an isolate the cells showing the mismatch and isolate ID are highlighted in grey.

| **Run** | **Substitution Model** | **Clock Model** | **Population Model** | **Path Sampling** | **Stepping Stone Sampling** | **Converged** |
| --- | --- | --- | --- | --- | --- | --- |
| 1 | JC | Strict | Constant | -5629980.18 | -5629974.54 | YES |
| 2 | HKY | Strict | Constant | -5435731.80 | -5435724.63 | YES |
| 3 | GTR | Strict | Constant | -5435743.40 | -5435740.84 | YES |
|  |  |  |  |  |  |  |
| 4 | HKY | Relaxed-Log | Constant | - | - | NO |
| 5 | HKY | Relaxed-Exp | Constant | -5435687.14 | -5435685.90 | YES |
|  |  |  |  |  |  |  |
| 6 | HKY | Relaxed-Exp | Logistic | - | - | NO |
| 7 | HKY | Relaxed-Exp | Exponential | -5435682.54 | -5435681.21 | YES |
| 8 | HKY | Relaxed-Exp | Expansion | - | - | NO |
| 9 | HKY | Relaxed-Exp | Skyline | -5435682.32 | -5435681.25 | YES |
| 10 | HKY | Relaxed-Exp | Skyride | -5435692.02 | -5435690.30 | YES |
| 11 | HKY | Relaxed-Exp | Skygrid | -5435685.72 | -5435683.68 | YES |

Table S2: A hierarchical approach to model selection for the BEAST analyses. Each model structure described above (defined by the substitution, clock and population models selected) was repeated three times using a chain length of 500,000,000 with every 50,000 step being sampled. The average likelihood (across the three replicates) is reported for the Path Sampling and Stepping Stone model comparison methods. In addition, whether or not the replicates converged was reported.
